# Diffusion-based mechanism explains spatial organization in cross-feeding biofilms

**DOI:** 10.1038/s41522-025-00719-5

**Published:** 2025-06-11

**Authors:** Julio Pérez, Cristian Picioreanu

**Affiliations:** 1 Department of Chemical, Biological and Environmental Engineering, Universitat Autònoma de Barcelona, Campus UAB, 08193 Bellaterra, Barcelona Spain; 2 Biological and Environmental Science and Engineering Division (BESE), King Abdullah University of Science and Technology (KAUST), 23955-6900 Thuwal, Saudi Arabia

**Keywords:** Evolution, Microbiology

## Abstract

Complex symbiotic interactions were claimed for explaining spatial organization of microbial species in cross-feeding biofilms. Here however, a distinct mechanism is proposed, called diffusion-based enhanced microbial organization (DEMO). An accepted mathematical model based on one-dimensional balances with diffusion-reaction of substrates and convection of multiple microbial types in a cross-feeding biofilm was used to describe emerging microbial distributions. The model allowed isolation of the effects of diffusion from other factors (kinetics, stoichiometry, specific symbiotic interactions), pointing to a possible mechanism for stratification in anaerobic biofilms. The secondary degrader consuming waste metabolite from a primary degrader was retained in anaerobic biofilms in an apparent growth yield disproportion. However, diffusion of an intermediate substrate can be responsible for this disproportion, even in longer food chains. This microbial distribution was not observed in independent feeding. In aerobic biofilms, this mechanism remains inactive, explaining the preference for full oxidation of organic matter in aerobic degradation.

## Introduction

The majority of bacteria and archaea exist as biofilms in their natural habitats (ca. 80%)^1^. Metabolite cross-feeding (including syntrophy) is widespread in both anaerobic and aerobic strata of biofilms^1,2^. Metabolic pathways are often divided between different species. A first microorganism (primary degrader, or producer) partly catabolizes the primary substrate releasing a waste metabolite (secondary substrate, or intermediate) that can be further degraded by another microorganism (secondary degrader, or consumer). This chain can continue with further steps of intermediate conversions, performed by other microorganisms^3^.

Many examples are found in natural ecosystems (e.g., lake sediments) as well as engineered systems (e.g., biofilm reactors). Cross-feeding biofilms are important for human, and in general animal, health (e.g., gut microbiota, oral biofilms, dental plaque, microbial infections, or biofilms growing on implants). These attached microbial communities are involved in global biogeochemical cycling, and present a wide variety of biotechnological applications (anaerobic and aerobic granular sludge, in general biofilm reactors, energy production with microbial fuel cells, among many others)^1,2,4^. Examples, within the anaerobic digestion chain: primary degrader sugar into low-chain carboxylic acids (acidifiers), secondary degrader fatty acids into acetic acid (acetogens), tertiary degrader acetic acid to methane (methanogens)^5,6^. Another case involves a co-culture of *Desulfovibrio* fed with lactate (primary producer of hydrogen) and *Methanococcus* (secondary degrader, producing methane from H_2_ and CO_2_)^7^. Aerobically, nitrification is widely occurring in two steps when ammonia oxidizing bacteria (AOB) oxidize ammonia into nitrite and nitrite it is further oxidized to nitrate by nitrite-oxidizing bacteria (NOB) (among many others, see ref. ^8^). All these metabolic interactions in biofilms rely on transport of metabolites by diffusion.

Although the role of diffusion of solutes (substrates, intermediates, products, etc.) in biofilms has been extensively described^9,10^, the spatial organization in cross-feeding biofilms has been frequently explained either by kinetic competition (e.g., refs. ^5,6^) or by complex symbiotic relationships (e.g., signaling) evolved in these microbial consortia^11–18^. Chemical gradients have been recognized for decades to lead and result from physiological heterogeneity in biofilms (e.g., recently reviewed by Jo et al.^19^). In fact, those gradients are crucial for the emergence of different physiological states of the microbes and even phenotypical heterogeneity in a biofilm or even in a cell cluster or microcolony^19,20^. This heterogeneity is thought to be a source for the structural and functional emergent properties of biofilms^20,21^. However, to date, the isolation and independent evaluation of the combined effect of solute transport, chemical gradients and microbial distribution and abundance remains elusive for cross-feeding biofilms.

Following the early development of one-dimensional biofilm modeling^22^, numerical simulations have been intensively used to compare model outputs to sets of experimental data obtained from a wide variety of bioprocesses. However, an accurate measurement of the biomass amounts of each microbial species within the biofilm is not widespread in experimental studies where a direct comparison of simulations and experimental data was intended. The experimental measurement of the abundance of the specific groups of microorganisms have been identified as one of the bottlenecks for deepening in the understanding of mixed microbial processes^23^. There are well-established molecular techniques, such as FISH and amplicon sequencing, which allow the quantification of microbial species in biofilm reactors. However, applying these methods to obtain a biomass concentration profile across biofilm depth remains challenging. While quantitative PCR (qPCR) is effective for determining total microbial abundance, its application to spatially resolving biomass distribution within biofilms is still limited, making direct comparisons with model predictions difficult. The mechanistic nature of biomass localization and abundance remained unidentified, firstly because the research efforts were devoted to determine the set of parameters that offered a good description of the experimental substrate and product concentrations, without a direct comparison of the experimental and predicted individual biomass concentrations^24^. Secondly, the simultaneous effects of mass transfer (diffusion), stoichiometry and kinetics obscured the detection of a clear mechanism for microbial localization and abundance. By numerical models, however, it is possible to turn off processes otherwise impossible to switch off in experiments, thus study in separation the effects of individual factors.

In this study, we propose a theoretical explanation for the emergent spatial organization of metabolite cross-feeding biofilms, based on the quantitative diffusion-reaction principles embedded in a traditional one-dimensional biofilm model.

## Results

### Anaerobic biofilms

#### Cross-feeding vs. independent use of substrate

**Fig. 1:**
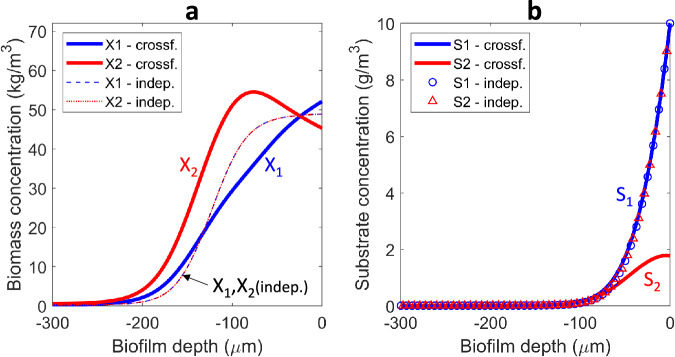
Anaerobic cross-feeding biofilm. Emerging (i.e., steady state) active biomass (**a**) and substrate (**b**) concentration profiles over the biofilm depth (water interface is at 0 μm), in anaerobic cross-feeding (*Case A1*) compared with anaerobic independent feeding (*Case A2*). The inert biomass concentration *X*_I_ is not plotted in (**a**), but it is the difference *X*_*I*_ = *X*_*f,tot*_ − *X*_1_ − *X*_2_, with *X*_*f,tot*_ = 100 kg COD/m^3^.

Model simulations made with a two-species anaerobic biofilm (*Case A1*) converged to a steady state, i.e., all system variables—concentrations of solutes and biomass in bulk liquid and biofilm reached constant values after several hundreds of days. All the model parameters are summarized in Supplementary Table 1. At steady state, the maximum concentration of the primary degrader *X*_1_ in the biofilm was at the biofilm surface (i.e., in contact with the water phase), while the secondary degrader *X*_2_ was relegated to deeper layers with a maximum concentration around 100 μm from the surface (Fig. 1a). The active biomass resided in a 200 μm layer, with deeper biofilm containing only inert material resulted from biomass decay. Interestingly, the secondary degrader was retained in the biofilm in a larger fraction than the primary degrader (overall, 26% X_2_, 19% X_1_, 55% inert). This resulted in a greater conversion of the intermediate in the reactor, compared with the primary substrate. The degree of conversion for the primary substrate is defined as (*S*_*in,1*_−*S*_*b,1*_)/*S*_*in,1*_ = (100−10)/100 which means 90% conversion. The degree of conversion for the secondary substrate has to take into account its availability when produced from *S*_1_, as if $(1-{Y}_{1})({S}_{in,1}-{S}_{b,1})$ = (1–0.1)(100−10) = 81 g/m^3^ *S*_2_ were produced without *S*_2_ degradation. These virtual 81 g/m^3^ *S*_2_ were converted down to ~2 g/m^3^ in the bulk liquid (Fig. 1b), which means (81−2)/81 ≈ 0.975 or ~97.5% degree of conversion.

For comparison, the steady state achieved in a two-species biofilm in which each microbial species catabolizes independent substrates (with the same stoichiometry and rates and same feeding from the liquid; *Case A2*) is also represented in Fig. 1a. When comparing the two strategies, it appears that the cross-feeding consortium makes better use of biofilm space, resulting in a greater total amount of active biomass. Furthermore, compared to the case of non-interacting microbial species, the cross-feeder occupied a larger region whereas the primary degrader was pushed closer to the biofilm surface (Fig. 1a). Microbial consortia growing in structured environments such as biofilms exhibit emergent properties, which are the properties not found when studying free-living (planktonic) cells^1^. The described set of differences found for cross-feeding anaerobic biofilms are indeed emergent properties.

This theoretical exercise produces an explanation on how the secondary degrader influences the location and abundance of the primary degrader, and what the consequences in terms of substrate conversion are.

#### Effect of primary substrate concentration

**Fig. 2:**
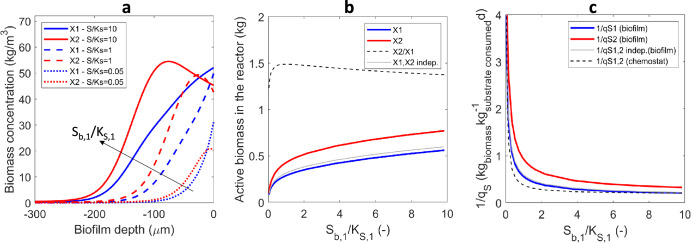
Effects of substrate concentration in bulk water on the emergent properties of an anaerobic cross-feeding biofilm (Case A1). **a** Spatial distributions of the two microbial types over the biofilm depth. **b** Total biomass (kg) of each type retained in the biofilm reactor, function of the primary substrate concentration in bulk liquid relative to the half-saturation coefficient (*S*_*b,*1_/*K*_*S,*1_). Ratio *X*_2_/*X*_1_ retained in the cross-feeding case also shows a greater abundance of the secondary degrader (by a factor 1.3 to 1.5). Cross-feeding was compared with independent feeding. **c** Ratio of total biomass retained in the reactor per total rate of substrate consumed in the biofilm. This is equivalent with an inverse of the biomass-specific substrate uptake rate, 1/*q*_*S*_, in kg biomass /(kg substrate consumed/d). For comparison, 1/*q*_*S*_ in unstructured environments (e.g., chemostats) was also plotted. Note how the changes in *S*_1,b_ have been depicted by using the *S*_1,b_/*K*_S,1_ ratio. This magnitude makes the effect of the changes in concentration dimensionless, independent on the particular *K*_S_ value selected and therefore more general.

The segregation of the two microbial populations in the biofilm (Fig. 2a), the higher proportion of secondary degrader (Fig. 2b) and its superior conversion degree were also obtained for a wide range of primary substrate concentrations in the bulk liquid *S*_*b*_ (represented here relative to the half-saturation coefficient *K*_*S*_). Eutrophic environments (i.e., high concentrations of the primary substrate) would favor stratification, in contrast to what would happen in oligotrophic environments (Fig. 2a). The total amounts of the two microbial species in the biofilm differed significantly (Fig. 2b), constituting an apparent growth yield disproportion. This can be explained by the better retention of the secondary degrader in the reactor per total rate of substrate consumed, i.e., kg biomass/(kg substrate consumed/d)(Fig. 2c). The apparent growth yield disproportion (quantified in the simulations as *m*_2_/*m*_1_) depends on the primary substrate concentration imposed in the bulk liquid (Fig. 2b). The maximum disproportion (ca. *m*_2_/*m*_1_ = 1.5, roughly 60% disproportion) was found at substrates concentration around the half-saturation coefficient (*S*_b,1_ ≈ *K*_S,1_). For eutrophic environments, when there is a high availability of primary substrate (e.g., *S*_b,1_/*K*_S,1_ = 10), and the stratification is enhanced (Fig. 2a), the disproportion is ca. 50% (Fig. 2b).

#### Effect of diffusion coefficients

**Fig. 3:**
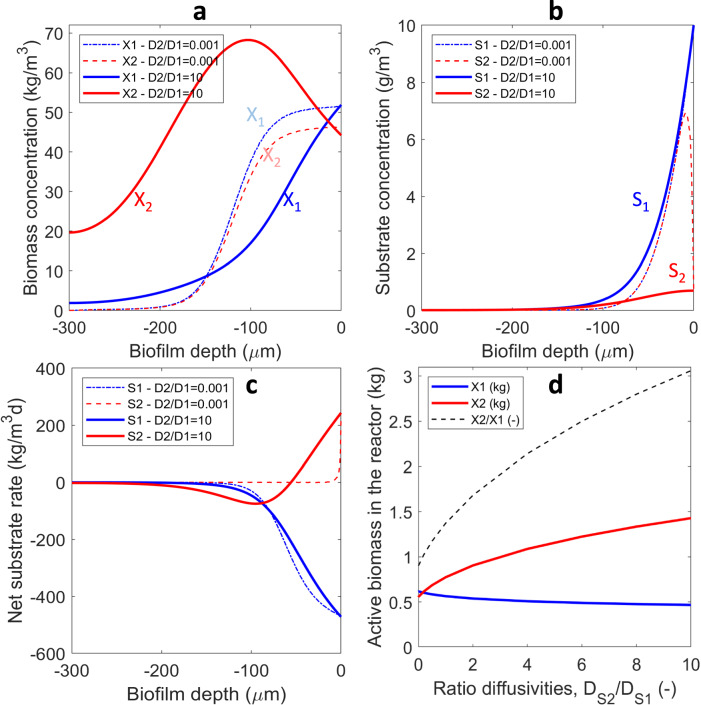
Anaerobic cross-feeding biofilm. Effect of diffusivity of secondary substrate on the emergent distribution of biomass concentrations (**a**), substrate concentrations (**b**) and net substrate rates (**c**) over the biofilm depth, for anaerobic cross-feeding biofilms (*Case A1*). **d** Total active biomass types retained in the reactor (kg biomass) function of relative diffusivity *D*_*S*2_/*D*_*S*1_. The ratio *X*_2_/*X*_1_ retained (dashed line) shows a much greater abundance of the secondary degrader with increasing *D*_*S*2_, while the theoretical ratio of 0.9 (i.e., 1−*Y*_1_, like in a chemostat) is attained when *D*_*S*2_ tends to zero.

The diffusion coefficients within the biofilm indicate how easily a compound moves through the biofilm. Large molecules (e.g., a protein) have a very small diffusion coefficient, even two orders of magnitude lower than that of H_2_, which is the smallest known molecule involved as substrate in anaerobic digestion. Because in general the diffusivities are expected to increase along the food chain (smaller products) several cases were considered in order to highlight the role of the diffusion of the intermediate (see Fig. 3). If the diffusion coefficient of the intermediate was set to a very low value (i.e., *D*_*S*2_ tends to zero), meaning that the intermediate is (almost) non-diffusive, both microbial species would have the maximum concentration at the surface of the biofilm and there is no trend towards stratification, Fig. 3a. In this case, the intermediate could not diffuse out of the biofilm and it is consumed on spot by the secondary degrader, with the additional effect of having almost no intermediate in the bulk liquid (Fig. 3b).

Plotting the distribution of net substrate rates in the biofilm offers more insights (Fig. 3c). While *S*_1_ can only be consumed in the biofilm (thus *r*_*S*1_ is always negative), because *S*_2_ can be produced by *X*_1_ and consumed by *X*_2_, the net rate *r*_*S*2_ can be positive or negative. Indeed, the net secondary substrate rate *r*_*S*2_ presents a minimum (that is, maximum *consumption* rate) at 100 μm from the surface, exactly at the position where the biomass concentration *X*_2_ is maximum as well. Closer to the surface *S*_2_ is mainly produced (positive net rate), but *S*_2_ consumption extends deeper in the biofilm due to its diffusion. This leads to an advantage for the secondary consumer *X*_2_, which can occupy more space in the biofilm. The diffusion of secondary substrate is a key factor in this emerging stratification: when *D*_*S*2_ is very much reduced (*D*_*S*2_/*D*_*S*1_ = 0.001) the *S*_2_ is consumed at the place of its production, leading to a net zero rate *r*_*S*2_ (see Fig. 3c) and no advantage for *X*_2_ (Fig. 3a). Note that when *S*_2_ is consumed on the spot, the total accumulated biomass *X*_2_ is 90% of *X*_1_ because according to the yield only 90% of *S*_1_ results in *S*_2_, exactly as it would happen in a chemostat. However, when *D*_*S*2_ was greater than ~0.1*D*_*S*1_ (for this set of parameters) the cross-feeding already results in an advantage of the secondary consumer. Compared to the first degrader, the cross-feeder reaches a higher abundance because its substrate (the intermediate *S*_2_) is produced in the biofilm instead of diffusing from the liquid.

#### Three-species cross-feeding

**Fig. 4:**
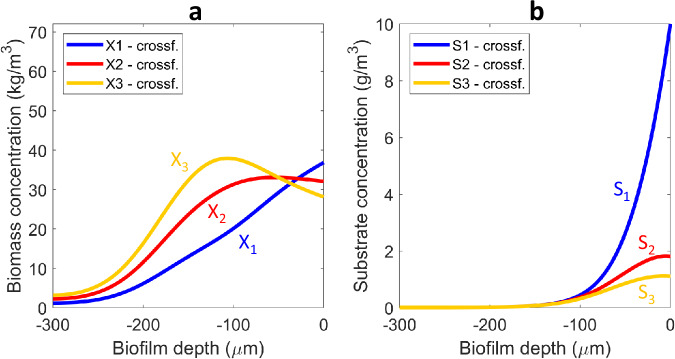
Three-species anaerobic corss-feeding biofilm. Emerging active biomass (**a**) and substrate (**b**) concentration profiles over the biofilm depth for a three-species anaerobic cross-feeding chain (*Case A3*: all reaction and transport parameters identical for all microbial types and solutes).

The anaerobic cross-feeding mechanism can be easily extended to longer food chains. Here we created a third step, performed in exactly the same way as the first two. The obtained results indicated how longer food chains shape the distribution of the three microbial species in the biofilm due to diffusion of intermediates, even with the same competing abilities (i.e., same growth rates and same growth yields). The existence of a third step in the degradation chain resulted in active biomass at deeper layers and the total active biomass retained in the biofilm (1.8 kg) was greater than for two species consortium (1.4 kg). Interestingly, the trend towards stratification between *X*_1_ and *X*_2_ holds (compared to the two steps food chain) whereas the last commensal in the food chain (*X*_3_) dominates the biofilm, because its substrate (*S*_3_) is produced in situ in the biofilm at deeper layers by *X*_2_ (Fig. 4). The disproportion in terms of active biomass amounts for each species holds comparing *X*_2_ and *X*_3_ as it was for *X*_1_ and *X*_2_ in the two steps food chain. Residual substrate concentration *S*_2_ is smaller than the residual concentration of *S*_1_ because of a higher conversion of *S*_2_, as in the two steps food chain. Additionally, due to the same reasons previously discussed for the two steps food chain, the residual concentration *S*_3_ is smaller than that of *S*_2_. The flowrate of substrate required to obtain 10 mg/L *S*_1_ in the liquid is 25 m^3^/d for the three steps food chain whereas 30 m^3^/d were required for the two-step chain. Thus, the longer the food chain, the slower the (overall) degradation rate achieved. This occurs because the amount of *X*_1_ in the biofilm is smaller.

### Aerobic heterotrophic biofilms

**Fig. 5:**
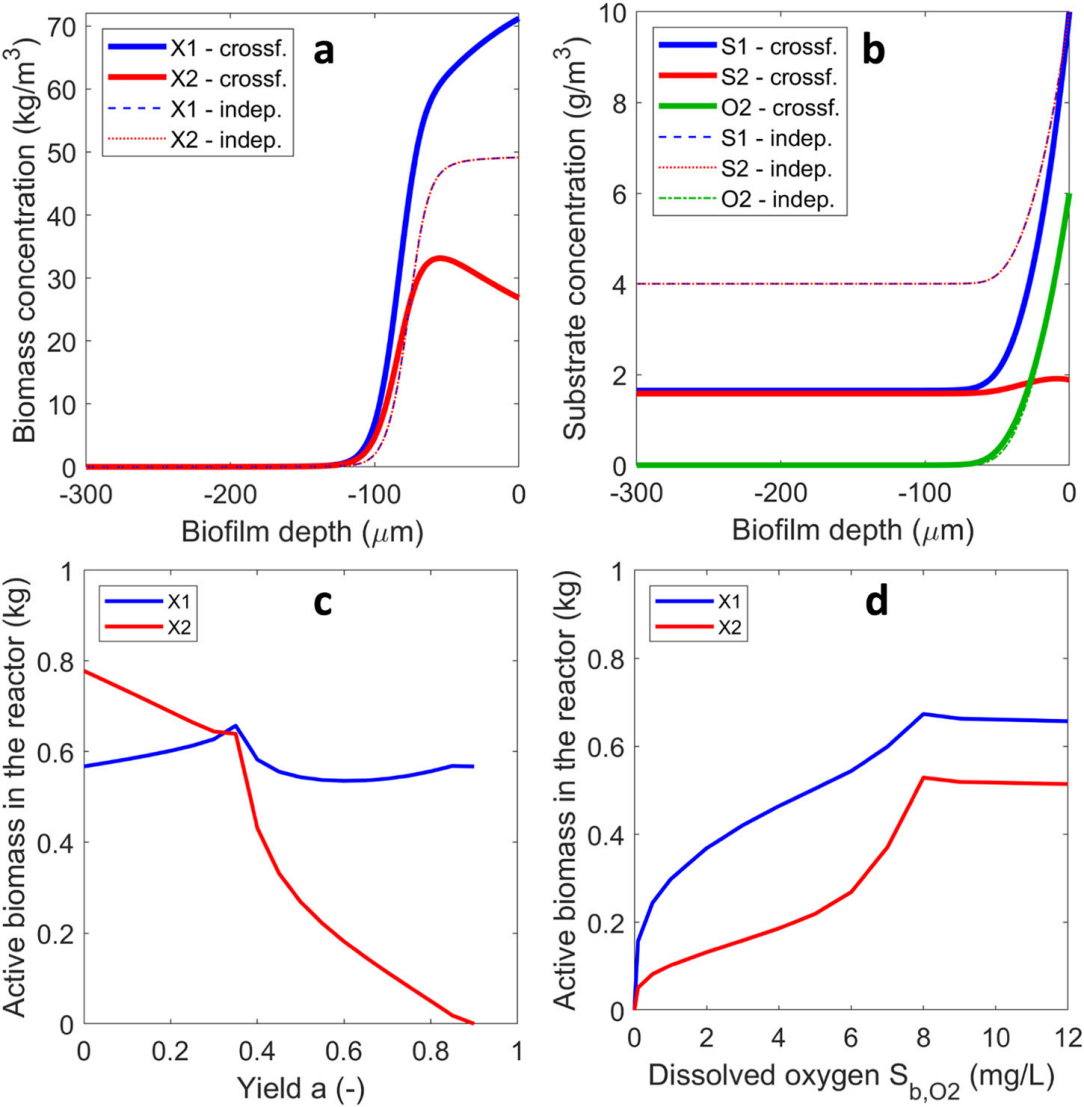
Emergent properties in aerobic heterotrophic cross-feeding biofilms (Case B1). Distribution of (**a**) microbial species and **b** solutes, in the aerobic biofilm. Effect of the yield on oxygen *a* (**c**) and the dissolved oxygen concentration (**d**) on the amounts of primary and secondary degrader retained in the biofilm. Note how abrupt the change is in active biomass at the switch from oxygen limitation to substrate limitation.

When the same cross-feeding reaction scheme was used, but both microbial species were respiring (i.e., aerobic biofilms with O_2_ required in addition to the organic substrates, *Case B1*), their interactions drastically changed. The competition narrowed the region available for substrate degradation within the oxygen penetration layer. When the oxygen penetration is shorter than that of substrates (Fig. 5b), then (1) there is no stratification and (2) the second degrader is maintained in smaller amounts in the biofilm (Fig. 5a). In spite of this apparent disadvantage of the second degrader, *X*_2_, the overall substrate conversion is very similar with that from the anaerobic case. However, this behavior can be changed if *X*_2_ has a better oxygen affinity, thus it will establish also in the deeper biofilm layers leading stratification (Supplementary Fig. 1).

We expect however that varying the DO concentration in the bulk or the yield *a* will have important effects on these emerging biofilm properties. In case of a primary substrate *S*_1_ not easily degradable, the yield *a* will be low, meaning that the energy left in *S*_2_ may be sufficient to restore the competitiveness of *X*_2_. Indeed, simulations clearly showed that below a ≈ 0.3 the secondary degrader *X*_2_ dominates the biofilm (Fig. 5c). When *a*→0 (i.e., little O_2_ utilization), the trends converge towards those observed in anaerobic biofilms, with *X*_2_ in larger amounts and clear stratification. Conversely, when the primary substrate is converted in more oxidized compounds, then *X*_1_ dominates, reaching complete extinction of *X*_2_ in the limit of *a* = 0.9—i.e., when the end product is CO_2_. Interestingly, the overall substrate utilization in the biofilm remains almost constant with varying *a* (Supplementary Fig. 2b). This can be due to the fact that *X*_2_ can be in a less favorable position in the biofilm, behind *X*_1_ (i.e., in deeper layers) but in larger amounts (as in the anaerobic case, *a* = 0), or in a favorable position close to the biofilm surface but in less amounts (as in the almost complete substrate oxidation case, *a*→0.9) (Supplementary Fig. 2a).

By increasing the DO concentration in the water (maintaining *a* = 0.5), the biofilm switches from oxygen limitation to substrate limitation (Supplementary Fig. 3b) above 8 mg DO/L. However, the primary degrader remains the dominant biofilm population (Fig. 5d) and the stratification is still minor (Supplementary Fig. 3a). Thus, in the studied conditions, the oxygen utilization fraction appears to be more influential than the DO levels in the liquid.

### Aerobic autotrophic biofilms

**Fig. 6:**
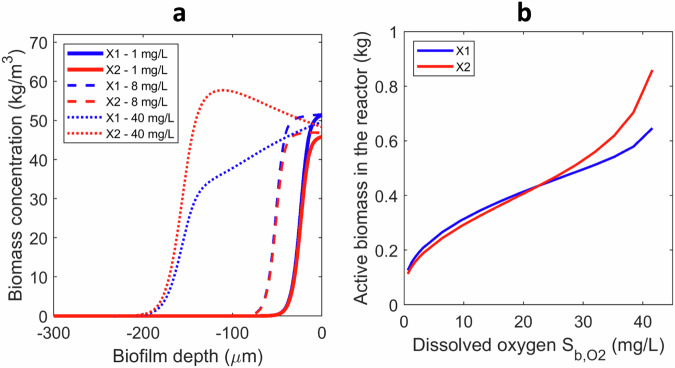
Emergent properties in aerobic autotrophic cross-feeding biofilms (Case B2). **a** Distribution of microbial species in the biofilm at three DO levels. **b** Total active biomass types retained in the reactor (kg biomass) function of DO concentration in the liquid.

In heterotrophic aerobic biofilms when a cross-feeding interaction settles, the yield on oxygen (determined by factor *a*) shapes the distribution and the abundances of the microbial species in the biofilm. When aerobic cross-feeding is autotrophic (e.g., the N usage in the aerobic nitrification food chain), the primary substrate is stoichiometrically converted in secondary substrate, and the role of oxygen differs (*Case B2*). Thus, from the substrate yield perspective, the secondary degrader should not be disadvantaged. In case of identical kinetics of the two microbial types, the poor solubility of oxygen in water prevented the cross-feeding emergent properties to arise in the aerobic strata of the biofilm below DO ~ 20 mg/L (Fig. 6b). However, for higher DO concentrations (e.g., at increased oxygen partial pressure) the cross-feeding emergent properties were activated with *X*_2_ getting dominant as in the anaerobic case (Fig. 6a).

When the secondary degrader has more affinity for oxygen (${K}_{O2,2}=0.25\,\cdot {K}_{O2,1}$), all discussed effects triggered by the diffusion of the intermediate would apply as in the anaerobic case (see Supplementary Fig. 4). In other words, a higher oxygen affinity of the cross-feeder activated the cross-feeding biofilm emergent properties.

## Discussion

To strengthen the connection between our theoretical findings and experimental evidence, we conducted specific simulations to describe microbial distribution and substrate concentration profiles in anaerobic biofilms degrading butyrate. In such biofilms and granules, microbial communities exhibit a well-defined stratified organization: (1) Outer layers (proximal to bulk liquid) are primarily occupied by fermentative butyrate degraders, typically *Syntrophomonas* spp., which oxidize butyrate into acetate, hydrogen (H_2_), and carbon dioxide (CO_2_)^25^; (2) Middle layers contain hydrogenotrophic and acetoclastic methanogens, belonging to the *Methanothrix* genus, which consume acetate to produce methane (CH_4_)^25^; (3) Inner core (deepest layers) may harbor sulfate-reducing bacteria and slow-growing methanogens, oxidizing butyrate while reducing sulfate to hydrogen sulfide (H_2_S)^26^. The study by Ziels et al.^25^ utilized DNA stable-isotope probing (DNA-SIP) with [¹³C]-butyrate to trace active butyrate degraders in anaerobic digesters. By applying genome-resolved metagenomics, the study reconstructed microbial genomes from labeled DNA, followed by differential abundance analysis to identify enriched taxa. Phylogenomic analysis determined the taxonomy of syntrophic bacteria, while metabolic pathway reconstruction inferred their butyrate degradation and interspecies electron transfer capabilities. These methods provided a comprehensive view of microbial community structure and function in syntrophic butyrate metabolism.

When anaerobic biofilms are fed a more complex organic substrate, experimental evidence further supports a layered microbial distribution. However, an additional outer layer of bacteria emerges, responsible for the hydrolysis of complex organic compounds and the fermentation of sugars and proteins into volatile fatty acids (VFAs), hydrogen (H_2_), and carbon dioxide (CO_2_). This stratification has been extensively documented in studies such as Satoh et al.^27^, Araujo et al.^28^ and Lee et al.^29^. These studies employed fluorescence in situ hybridization (FISH) combined with confocal laser scanning microscopy (CLSM) to reveal the spatial organization and functional roles of microbial communities in anaerobic biofilms, particularly those involved in butyrate metabolism.

**Fig. 7:**
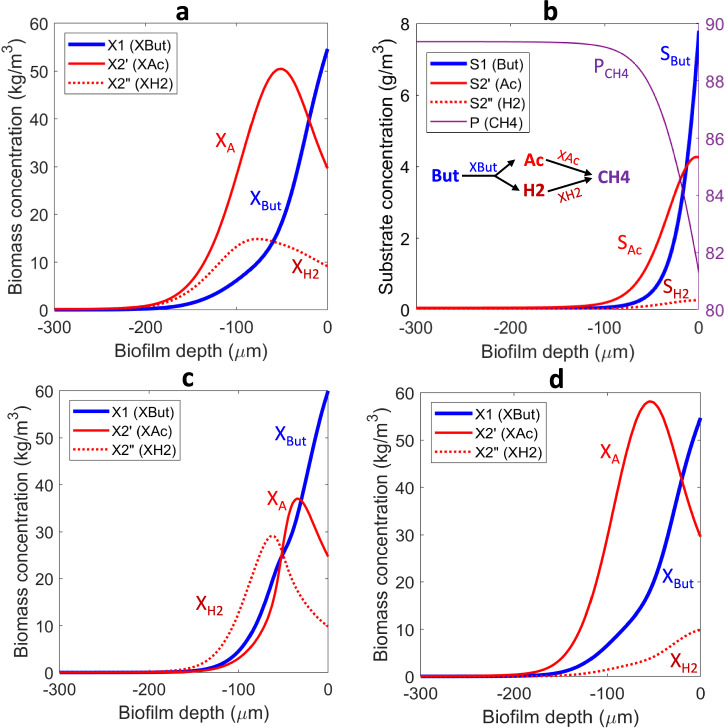
Butyrate fed to anaerobic biofilm as real case scenario. Microbial distribution (**a**) and substrate distribution (**b**) in the biofilm for the anaerobic degradation of butyrate (*S*_1_) to acetate (*S*_2_’) and hydrogen (*S*_2_”) by organism *X*_1_, followed by methanogenesis by aceticlastic (*X*_2_’) and hydrogenotrophic (*X*_2_”) organisms in an anaerobic biofilm fed with butyrate. Product (CH_4_) concentration (g/m^3^) is shown on the right *y*-axis of (**b**). Simulations used the kinetics, stoichiometry and parameters described in Supplementary Table 2. Microbial distribution in the biofilm when acetate (*S*_2_’) is considered (almost) non-diffusive (${D}_{2}^{\text{'}}=0.01\cdot {D}_{2,basecase}^{\text{'}}$) (**c**). Microbial distribution in the biofilm when H_2_ (*S*_2_”) is considered (almost) non-diffusive (${D}_{2}^{\text{'}\text{'}}=0.01\cdot {D}_{2,basecase}^{\text{'}\text{'}}$) (**d**).

To simplify this complex system for our model, we focused on primary and secondary degraders only. The anaerobic degradation of butyrate (*S*_1_) into acetate (*S*_2_’) and hydrogen (*S*_2_”) is performed by organism *X*_1_, followed by methanogenesis by aceticlastic (*X*_2_’) and hydrogenotrophic (*X*_2_”) organisms, as depicted in Fig. 7b and Supplementary Table 2. The model results align with our main findings on anaerobic biofilms and the experimental evidence reported in previous studies. Indeed, the secondary degraders (the methanogens *X*_2_’ and *X*_2_”) thrive in intermediate layers, deeper in the biofilm than the primary butyrate degraders (*X*_1_). The hydrogenotrophs *X*_2_” develop in smaller amounts than the aceticlastic methanogens *X*_2_’ because butyrate is fermented in only 20% H_2_ versus 80% acetate (COD based) (Fig. 7a). Also, in agreement with the microsensor solute concentration measurements of Satoh et al.^27^, a peak of H_2_ forms near the biofilm surface, while the CH_4_ accumulates in the biofilm depth and continuously decreases towards the biofilm surface.

To further highlight the role of intermediate diffusion on emergent biofilm properties, we independently reduced the diffusion coefficients of acetate and H_2_ drastically, by a factor of one hundred. The simulations demonstrated that slower diffusion led to reduced stratification and decreased disproportion between microbial groups (Fig. 7c, d), thus emphasizing the crucial role of diffusion on microbial distribution in biofilms.

In the literature, higher conversion of resources and higher biomass productivity have been described as emergent properties of biofilms^12–15,18,30,31^. The mechanism proposed in our study, based on the diffusion of an intermediate produced in the anaerobic biofilm, can shape: (1) the extent of stratification of microbial species (i.e., the species distribution over biofilm depth, Figs. 2a and 3a); (2) the proportion between species in the biofilm at steady state (i.e., the microbial abundance) (Fig. 3d); (3) the amount of total active biomass (Figs. 2b and 3d) because the secondary degrader *X*_2_ is retained in a higher concentration; (4) the slightly decreased overall primary substrate consumption rate (Fig. 2c), while having a better conversion of the intermediate. The distinct growth of the secondary degrader in a deeper layer compared with the primary degrader is in this mechanism mediated by a physical trigger: the diffusion of the intermediate. All these demonstrate that microbial consortia growing in anaerobic biofilms exhibit specific emergent properties, which are not found when studying free-living (planktonic) cells. Certainly, the biofilm-specific mechanisms might be amplified or reduced by varying the kinetic and stoichiometric parameters of the microorganisms involved, the specific ecological interactions or the substrate transport rates (e.g., diffusivities). We deliberately excluded from this mathematical model the impact of different stoichiometry and kinetics of the interacting species, and specific symbiotic interactions. Consequently, we believe that studies in which stoichiometry, kinetics and/or specialized microbial interactions have been considered as a prime factor to explain anaerobic biofilm architecture, higher conversion of the intermediate and increased biomass retention^5,6,11–18^ need some reconsideration.

The set of cross-feeding emergent properties found for anaerobic biofilms may constitute a driving force for the evolution of division of labor, because the mechanism favors the evolution towards short pathways leading to incomplete oxidation of the substrate. This can be proven by enlarging the simulated microbial consortium with one more microorganism (${X}_{1}^{\ast }$) that can degrade the primary substrate *S*_1_ directly to the end product *P*. This full degrader microbe may have a larger yield (${Y}_{1}^{\ast }$> ${Y}_{1}$), but a slower maximum specific growth rate (${\mu }_{m,1}^{\ast }$< ${\mu }_{m,1}$), according to the kinetic theory of optimal pathway length^8,32^ and other experimental evidence^33^. Indeed, our simulations (Supplementary Fig. 5) indicate that the full degrader ${X}_{1}^{\ast }$ will be eventually outcompeted by the *X*_1_/*X*_2_ consortium when ${\mu }_{m,1}^{\ast }$ is only marginally smaller than ${\mu }_{m,1}$ (e.g., ${\mu }_{m,1}^{\ast }=0.95{\mu }_{m,1}$). However, the diffusion-based selection mechanism appears to be deactivated when the heterotrophic cross-feeding biofilm is under aerobic conditions (Fig. 5). This explains why the metabolic division of labor in organic matter decomposition is carried out into several steps by different types of microbes, typically for anoxic environments, but not for the aerobic ones. This question was highlighted and the mechanisms potentially responsible were theoretically analyzed by Kreft et al.^34^. However, none of the reasons put forward in ref. ^34^ included a direct link to the diffusivity of an intermediate. In our view, we propose here a fundamental mechanism, which could easily explain why a set of microbial groups can perform individual steps in the degradation chain of organic matter in anaerobic biofilms, but not in aerobic conditions.

When the biofilm is aerobic but colonized by autotrophic microbes (e.g., nitrification), the mechanism is also inactivated unless the secondary degrader (i.e., NOB) has a better oxygen affinity compared to the primary degrader (i.e., AOB). Indeed, a smaller intrinsic oxygen half- saturation coefficient has been mostly reported for NOB (see a compilation of values in ref. ^35^). Therefore, stratification of nitrifying guilds have been found stable in engineered ecosystems, with AOB occupying the external shell and NOB relegated to deeper layers (in granular sludge reactors, see ref. ^36^).

The importance of the newly proposed mechanism leading to biofilm emerging properties is of relevance for microbial ecology. It is widely recognized that mixed-species biofilms are characterized by heterogeneity and social interactions (see an overview in ref. ^30^). Biofilms are considered to be self-organized, and often the causes for a particular microbial organization (location and abundance of each of the microbial groups) have been linked to the excretion of signaling molecules, kinetic traits of the microbial groups (acquired in the course of natural selection) or even due to changes at genetic level (e.g., mutations). However, the mechanism here described linked to the diffusivity of the intermediate is a passive driving force for biofilm organization, without the need to invoke complex reasons or even “self-organization”.

The scarcity of studies investigating the role of diffusion of the intermediate in cross-feeding biofilms led to the following assumption: the ratio of the primary degrader to secondary degrader (i.e., cross-feeder) retained in the biofilm is mainly conditioned by the growth yields (when assuming similar diffusion coefficients for substrate and intermediate)^35–38^. The assumption originates from growth measurements of planktonic cells in chemostats, for independent degradation pathways of S_1_ and S_2_ (see Table 1). However, the direct application of growth yield ratios to cross-feeding biofilms has been questioned, based on experimental evidence^14^. When an unexpectedly high proportion of cross-feeder has been experimentally found in syntrophic biofilms, complex (i.e., not yet understood) microbial interactions were claimed as an explanation^12,14^. For a co-culture of *Desulfovibrio vulgaris* (first commensal, fed with lactate, producing hydrogen) and *Methanococcus maripaludis* (cross-feeder producing methane using hydrogen and carbon dioxide), a syntrophic biofilm reactor was characterized by measuring the proportion of each one of the species and comparing it to the case of a planktonic co-culture^14^. The measured proportions of primary to secondary degrader changed from 6.3:1 (planktonic cells in chemostat) to 2.2:1 (growing as biofilm). This experimental evidence aligns with the model results from the anaerobic biofilm case (Fig. 1a), indicating a reduction in the proportion of primary degrader when the consortium approaches steady state. The authors in ref. ^14^ claimed that non-identified genetic and metabolic changes resulted in the enhanced proportion of second degrader, however, the observations could easily be explained as cross-feeding emergent properties.

**Table 1:** Generalization of emergent properties in cross-feeding anoxic biofilms compared to independent feeding biofilms and unstructured environments (i.e., chemostat)

Environment	Microbial interaction		Substrate conversion x (resource usage)	Biomass retention m	Substrate consumption-specific biomass retention $\frac{1}{{q}_{S}}=\frac{{\rm{biomass}}\,{\rm{retained}}}{{\rm{substrate}}\,{\rm{consumed}}/{\rm{time}}}$
Unstructured (chemostat, *Ch*)	Independent (*ind*)		${x}_{1}^{{Ch},{ind}}={x}_{2}^{{Ch},{ind}}$	${m}_{1}^{{Ch},{ind}}={m}_{2}^{{Ch},{ind}}$	${\left[\frac{1}{{q}_{S}}\right]}_{X1}^{{Ch},{ind}}={\left[\frac{1}{{q}_{S}}\right]}_{X2}^{{Ch},{ind}}$
	Cross-feeding (*cross*)		${x}_{2}^{{Ch},{cross}} > \,{x}_{1}^{{Ch},{cross}}$	${m}_{2}^{{Ch},{cross}} > \,{m}_{1}^{{Ch},{cross}}$	${\left[\frac{1}{{q}_{S}}\right]}_{X1}^{{Ch},{cross}}={\left[\frac{1}{{q}_{S}}\right]}_{X2}^{{Ch},{cross}}$
Structured (biofilm, *Bf*)	Independent (*ind*)		${x}_{1}^{{Bf},{ind}}={x}_{2}^{{Bf},{ind}}$	${m}_{1}^{{Bf},{ind}}={m}_{2}^{{Bf},{ind}}$	${\left[\frac{1}{{q}_{S}}\right]}_{X1}^{{Bf},{ind}}={\left[\frac{1}{{q}_{S}}\right]}_{X2}^{{Bf},{ind}}$
	Cross-feeding (*cross*)	Primary degrader (*X*_*1*_)	${x}_{1}^{{Bf},{cross}} < \,{x}_{1}^{{Bf},{ind}}$	${m}_{1}^{{Bf},{cross}} < \,{m}_{1}^{{Bf},{ind}}$	${\left[\frac{1}{{q}_{S}}\right]}_{X1}^{{Bf},{cross}} < {\left[\frac{1}{{q}_{S}}\right]}_{X1}^{{Bf},{ind}}$
		Secondary degrader (*X*_*2*_)	${x}_{2}^{{Bf},{cross}}\gg \,{x}_{2}^{{Bf},{ind}}$	${m}_{2}^{{Bf},{cross}}\gg \,{m}_{2}^{{Bf},{ind}}$	${\left[\frac{1}{{q}_{S}}\right]}_{X2}^{{Bf},{cross}}\gg {\left[\frac{1}{{q}_{S}}\right]}_{X2}^{{Bf},{ind}}$

Substrate conversion degree (resource utilization) *x*, biomass retention *m* and substrate consumption-specific biomass retention 1/*q*_*S*_ are defined in Supplementary Information (section Performance indicators). The analysis considers all kinetic parameters for degradation of substrate S_1_ by biomass X_1_ are the same with those for degradation of S_2_ by X_2_. Superscripts are *Ch* for chemostat, *Bf* for biofilm, *ind* for independent feeding, and *cross* for cross-feeding; Subscripts refer to biomass/substrate 1 or 2.

More support for the diffusion-based enhanced microbial organization appears from the study of substrate concentration microprofiles in the biofilm. The computed concentration profile for the intermediate substrate (*S*_2_) in the biofilm exhibits a maximum close to the biofilm surface (Fig. 1b). This maximum has been measured experimentally in several cases. First, the aerobic autotrophic cross-feeding biofilms constituted by AOB and NOB can display a maximum on nitrite (NO_2_^−^) concentration close to the biofilm surface, as measured in ref. ^39^ (in this case, ammonium is *S*_1_ and nitrite is *S*_2_). The model also predicts this maximum of *S*_2_ (Supplementary Fig. 4c). Also in a denitrifying biofilm, a maximum of NO_2_^−^ was measured (e.g., ref. ^40^), in agreement with the results obtained with the simulations (here, nitrate is *S*_1_ and nitrite is *S*_2_). Note that the maximum of intermediate *S*_*2*_ concentration appears very close to the surface (Fig. 1b) and it is not easily detectable when *D*_*1*_ = *D*_*2*_, but it is more pronounced when *D*_*2*_ < *D*_*1*_ (Fig. 3b). Nevertheless, this substrate maximum can lead to the development of microbial layers, which are easier to detect through microscopy (e.g., refs. ^39,41^). When the intermediate is present in the bulk liquid in higher concentrations than in the biofilm (e.g., massively produced by planktonic cells), it will diffuse in the biofilm. For such case, the flux of the intermediate would be exclusively directed towards the biofilm depth (i.e., no maximum in the intermediate concentration can appear), resulting in the deactivation of the driving force for microbial stratification in the biofilm.

While our study provides a theoretical framework for the diffusion-based enhanced microbial organization (DEMO) mechanism and its role in cross-feeding biofilms, several limitations should be acknowledged.

1. Simplifications in kinetics and stoichiometry: in order to enable the isolation of the diffusional effects, we set identical kinetic and stoichiometric parameters for all microbial species in each scenario. Obviously, in real biofilms, microorganisms exhibit different specific growth rates, substrate affinities, and yield coefficients. These variations could enhance or diminish the effects of the DEMO mechanism, depending on how substrate utilization dynamics interacts with diffusion-driven spatial organization. Future studies could explore in more detail how different kinetic and stoichiometric parameters influence biofilm stratification and microbial retention, beyond the effects of DEMO mechanism.
2. Assumptions on diffusion coefficients: with this model we explored the effect of diffusion coefficients by systematically varying them, but real biofilms exhibit heterogeneous and dynamic diffusion properties due to variations in extracellular polymeric substances (EPS) composition and biofilm density. Reduced (effective) diffusivity in deeper biofilm layers may also limit the strength of the DEMO mechanism, particularly for larger molecules with inherently low diffusivity.
3. Exclusion of additional ecological interactions: this theoretical study simply focuses on the physical role of diffusion and does not include specific microbial interactions beyond substrate diffusion, such as synergistic metabolic exchanges, product inhibition (e.g., H_2_) and symbiotic interactions^42^, or even quorum sensing^11–18^. These factors may modulate or compete with the effects of the DEMO mechanism in natural biofilms.

By acknowledging these limitations, we aim to guide future research efforts toward refining and experimentally validating the DEMO mechanism under more complex and realistic biofilm conditions.

A new and distinct mechanism is proposed in this study, which we called diffusion-based enhanced microbial organization (DEMO mechanism). By this mechanism, triggered by the diffusion of an intermediate substrate, the cross-feeder can be retained in the biofilm at amounts that reveal an apparent growth yield disproportion. By using a widely accepted one-dimensional biofilm model, we demonstrate that DEMO mechanism drives stratification of microbial species, enhances resource usage, and boosts biomass retention in anoxic biofilms (as summarized in Table 1). A generalization of the DEMO mechanism to represent longer food chains led to stratified microbial type distributions with outer layers dominated by primary degraders, going to inner layers made by further degraders - maintaining the order of food degradation steps. This microbial spatial segregation was not observed in independent feeding. We also showed that in case of aerobic biofilms the DEMO mechanism remains inactive, explaining the preference for full oxidation of organic matter in aerobic degradations. We believe the DEMO mechanism can be a passive driving force for the biofilm organization and microbial division of labor, mainly active in anoxic environments.

## Methods

### Biofilm model

The biofilm model used in this study is based on the traditional one-dimensional approach formulated by Wanner and Gujer^22^, which was included in the Aquasim software^43^. However, we implemented the mathematical model in a more modern environment, *COMSOL Multiphysics 6.1* (www.comsol.com). Model details including all equations are presented in the Supplementary Note 1, while here we briefly explain only the main model assumptions and features. Following the well-accepted methodology in modeling environmental engineering applications^44^, the system contains soluble components, S (e.g., substrates and oxygen), and particulate components, X (e.g., active biomass and inert material). These components participate in a series of conversion processes, described in the following paragraphs for each modeled case.

**Fig. 8:**
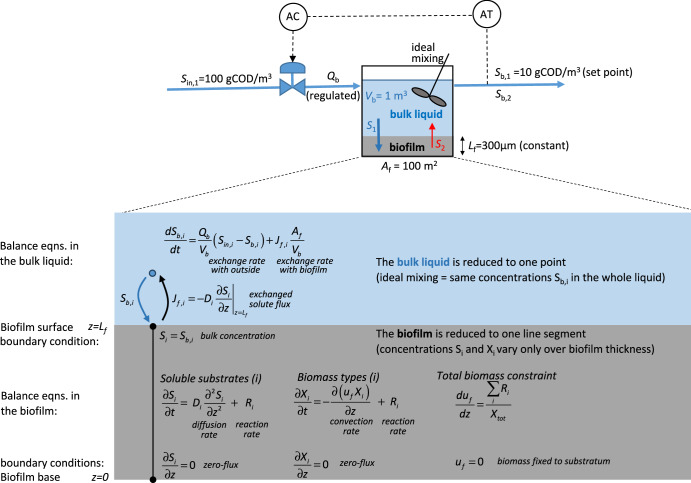
Schematic representation of the model. The top figure represents a continuous stirred tank reactor with a planar biofilm in which a closed control loop maintains constant the concentration of primary substrate in the bulk liquid (*S*_b,1_) by regulating the inflow rate (*Q*_b_) (AT and AC are analysis transmitter and controller, respectively). The bottom figure, details the biofilm model with its corresponding balance equations and boundary conditions, and their related meaning. Practically, this construction is very similar with the dynamic one-dimensional biofilm model developed by Wanner and Gujer^19^. The model was run for a long-enough time interval (e.g., 3 years) to reach the steady state microbial distribution in the biofilm, *X*_*i*_(*z*), the concentrations of substrates in the biofilm, *S*_*i*_(*z*), and the concentration of intermediate metabolite *S*_b,2_ in the bulk liquid (equal with that in the reactor effluent).

In the biofilm, concentration gradients for both solutes and particulates develop in one direction from the biofilm base to the biofilm interface with water. The time-dependent balances for soluble components in the biofilm include solute transport by diffusion only and source terms representing the reaction rates. There was co-diffusional transport of solutes, all being supplied from the liquid. The balances for particulate components in the biofilm consider transport by convection and source terms due to biomass growth and decay. It is essential that each microbial type exists initially in the biofilm. We chose a uniform initial biomass distribution throughout the biofilm, with equal fractions of each microbial type (although, the exact initial fraction did not matter in obtaining the same stationary distribution). Assuming that there is a maximum cell density (packing) in the biofilm, growing microbial cells must push other cells. This will generate movement of biomass from the biofilm depth towards the biofilm surface, with a certain velocity, which supports the biomass convection. The biomass convection velocity, varying over the biofilm thickness and in time, can be computed from a total biomass balance after imposing the constraint that the total biomass density must remain constant (see Supplementary Information Note 1 for a detailed explanation of the governing equations). The assumption of a constant maximum biomass concentration practically means that a fast-growing microbial type will replace the slower growing one, in a kind of competition for space within the biofilm. Therefore, there is a continuous movement of biomass from the biofilm depth towards the surface, where eventually the cells will be detached (and become planktonic) if a constant biofilm thickness is imposed. The biofilm phase is in contact with an aqueous phase (bulk liquid), representing a continuous ideally mixed biofilm reactor at constant liquid volume, exchanging solutes with the biofilm. We deliberately did not consider the activity of planktonic cells because that would complicate the analysis of the effects of substrate diffusion on the microbial distribution within the biofilm. The model can achieve a steady state, with solute and biomass gradients formed along the biofilm thickness and certain concentrations reached in the bulk liquid. See Fig. 8 for a diagram representation of the model.

### Microbial interaction cases

#### A. Anaerobic biofilms (no respiration, organic electron donor and acceptor)

##### Case A1: cross-feeding

As a simple and representative example of cross-feeding, consider the following sequential reactions performed by two microbial species:

1 $${S}_{1}\mathop{\longrightarrow}\limits^{{X}_{1}}_{\begin{array}{l}primary\\ degrader\end{array}}\,{S}_{2}\mathop{\longrightarrow}\limits^{{X}_{2}}_{\begin{array}{c}secondary\\ degrader\end{array}}\ldots$$

where primary substrate (*S*_1_) and secondary substrate (intermediate *S*_2_) are the main energy sources of the primary (*X*_1_) and the secondary (cross-feeder *X*_2_) degrader, respectively. When such a reaction scheme occurs in a biofilm, the access to the substrate differs for each microbial species. For the primary degrader the substrate diffuses only from the bulk liquid towards the biofilm depth. For the cross-feeder, the substrate is the intermediate compound *S*_2_, produced in situ by *X*_1_, which can diffuse both to the biofilm depth but also leak out into the surrounding water. The model included growth and decay for each biomass type. To assess the influence of this distinct access to the substrate, numerical simulations were carried out in which the growth kinetics (rate parameters *μ*_*m*_ and *K*_*S*_) and stoichiometry (yields *Y*) of both microbial species were identical and respecting the COD balances (Table 2). The decay rate coefficients *b* were also identical, the decay process meaning that the growing (active) biomass is converted into inert (inactive) particulate material—having the same density in the biofilm as the active cells.

**Table 2:** Stoichiometry and kinetics of the two-species community

Component →	S_1_	S_2_	P	O_2_	X_1_	X_2_	X_I_	Rate
Process ↓	Primary substrate	Secondary substrate	End product	Dissolved oxygen	Primary degrader	Secondary degrader	Inert biomass	
Case A1. Anaerobic cross-feeding ${S}_{1}\mathop{\longrightarrow }\limits^{{X}_{1}}\,{S}_{2}\mathop{\longrightarrow }\limits^{{X}_{2}}P$								
1. Growth *X*_*1*_	$-\frac{1}{{Y}_{1}}$	$\frac{1-{Y}_{1}}{{Y}_{1}}$			1			${\mu }_{m,1}\frac{{S}_{1}}{{K}_{S,1}+{S}_{1}}{X}_{1}$
2. Decay *X*_*1*_					-1		1	${b}_{1}{X}_{1}$
3. Growth *X*_*2*_		$-\frac{1}{{Y}_{2}}$	$\frac{1-{Y}_{2}}{{Y}_{2}}$			1		${\mu }_{m,2}\frac{{S}_{2}}{{K}_{S,2}+{S}_{2}}{X}_{2}$
4. Decay *X*_*2*_						-1	1	${b}_{2}{X}_{2}$
Case A2. Anaerobic independent feeding ${S}_{1}\mathop{\longrightarrow }\limits^{{X}_{1}}\,P;\,{S}_{2}\mathop{\longrightarrow }\limits^{{X}_{2}}P$								
1. Growth *X*_*1*_	$-\frac{1}{{Y}_{1}}$		$\frac{1-{Y}_{1}}{{Y}_{1}}$		1			${\mu }_{m,1}\frac{{S}_{1}}{{K}_{S,1}+{S}_{1}}{X}_{1}$
2. Decay *X*_*1*_					-1		1	${b}_{1}{X}_{1}$
3. Growth *X*_*2*_		$-\frac{1}{{Y}_{2}}$	$\frac{1-{Y}_{2}}{{Y}_{2}}$			1		${\mu }_{m,2}\frac{{S}_{2}}{{K}_{S,2}+{S}_{2}}{X}_{2}$
4. Decay *X*_*2*_						-1	1	${b}_{2}{X}_{2}$
Case B1. Aerobic heterotrophic cross-feeding ${S}_{1}\mathop{\longrightarrow}\limits^{X_{1}}_{O_{2}^{\nearrow}}\, {S}_{2}\mathop{\longrightarrow}\limits^{X_{2}}_{O_{2}^{\nearrow}}\,P$								
1. Growth *X*_*1*_	$-\frac{1}{{Y}_{1}}$	$\frac{1-{Y}_{1}-{a}_{1}}{{Y}_{1}}$		$-\frac{{a}_{1}}{{Y}_{1}}$	1			${\mu }_{m,1}\frac{{S}_{1}}{{K}_{S,1}+{S}_{1}}\frac{{S}_{O2}}{{K}_{O2,1}+{S}_{O2}}{X}_{1}$
2. Decay *X*_*1*_					-1		1	${b}_{1}{X}_{1}$
3. Growth *X*_*2*_		$-\frac{1}{{Y}_{2}}$	$\frac{1-{Y}_{2}-{a}_{2}}{{Y}_{2}}$	$-\frac{{a}_{2}}{{Y}_{2}}$		1		${\mu }_{m,2}\frac{{S}_{2}}{{K}_{S,2}+{S}_{2}}\frac{{S}_{O2}}{{K}_{O2,2}+{S}_{O2}}{X}_{2}$
4. Decay *X*_*2*_						-1	1	${b}_{2}{X}_{2}$
Units	$\frac{g\,COD}{{m}^{3}}$	$\frac{g\,COD}{{m}^{3}}$	$\frac{g\,COD}{{m}^{3}}$	$\frac{g\,{O}_{2}}{{m}^{3}}$	$\frac{g\,COD}{{m}^{3}}$	$\frac{g\,COD}{{m}^{3}}$	$\frac{g\,COD}{{m}^{3}}$	$\frac{g\,COD}{{m}^{3}d}$
Case B2. Aerobic autotrophic cross-feeding (e.g., nitrification) ${S}_{1}\mathop{\longrightarrow}\limits^{X_{1}}_{O_{2}^{\nearrow}}\, {S}_{2}\mathop{\longrightarrow}\limits^{X_{2}}_{O_{2}^{\nearrow}}\,P$								
1. Growth *X*_*1*_	$-\frac{1}{{Y}_{1}}$	$\frac{1}{{Y}_{1}}$		$-\frac{{\alpha }_{1}-{Y}_{1}}{{Y}_{1}}$	1			${\mu }_{m,1}\frac{{S}_{1}}{{K}_{S,1}+{S}_{1}}\frac{{S}_{O2}}{{K}_{O2,1}+{S}_{O2}}{X}_{1}$
2. Decay *X*_*1*_					-1		1	${b}_{1}{X}_{1}$
3. Growth *X*_*2*_		$-\frac{1}{{Y}_{2}}$	$\frac{1}{{Y}_{2}}$	$-\frac{{\alpha }_{2}-{Y}_{2}}{{Y}_{2}}$		1		${\mu }_{m,2}\frac{{S}_{2}}{{K}_{S,2}+{S}_{2}}\frac{{S}_{O2}}{{K}_{O2,2}+{S}_{O2}}{X}_{2}$
4. Decay *X*_*2*_						-1	1	${b}_{2}{X}_{2}$
Units	$\frac{g\,N}{{m}^{3}}$	$\frac{g\,N}{{m}^{3}}$	$\frac{g\,N}{{m}^{3}}$	$\frac{g\,{O}_{2}}{{m}^{3}}$	$\frac{g\,COD}{{m}^{3}}$	$\frac{g\,COD}{{m}^{3}}$	$\frac{g\,COD}{{m}^{3}}$	$\frac{g\,COD}{{m}^{3}d}$

Standard parameter values: maximum specific growth rate ${\mu }_{m,1}={\mu }_{m,2}=1$ d^−1^, decay rate coefficient ${b}_{1}={b}_{2}=0.01$ d^−1^, half-saturation coefficients for substrate ${K}_{S,1}={K}_{S,2}=1$ gCOD m^−3^ and biomass growth yields on substrate ${Y}_{1}={Y}_{2}=0.1$ gCOD-X/gCOD-S. In the aerobic heterotrophic case, ${K}_{O2,1}={K}_{O2,2}=0.1$ gO_2_ m^−3^, ${a}_{1}={a}_{2}=0.5$, except when otherwise stated. In the aerobic nitrification case *α*_1_ = 3.45, *α*_2_ = 1.15 gO_2_ gN^−1^, and ${Y}_{1}={Y}_{2}=0.2$ gCOD-X/gN.

##### Case A2: independent feeding

The cross-feeding scheme was compared with two microorganisms that use independently their substrates (Eq. (2), Table 2). *S*_2_ was also supplied in the reactor feed, identically with *S*_1_.

2 $$\begin{array}{l}{S}_{1}\mathop{\longrightarrow}\limits^{{X}_{1}}_{\begin{array}{c}primary\\ degrader\end{array}}\ldots\\{S}_{2}\mathop{\longrightarrow}\limits^{{X}_{2}}_{\begin{array}{c}secondary\\ degrader\end{array}}\ldots\end{array}$$

##### Case A3: cross-feeding with a longer food chain

The cross-feeding scheme (*Case A1*) was readily extended with a third degrader *X*_3_ and its corresponding substrate *S*_*3*_, following the secondary degrader (Eq. (3)):

3 $${S}_{1}\mathop{\longrightarrow}\limits^{{X}_{1}}_{\begin{array}{l}primary\\ degrader\end{array}}\,{S}_{2}\mathop{\longrightarrow}\limits^{{X}_{2}}_{\begin{array}{c}secondary\\ degrader\end{array}}\,{S}_{3}\mathop{\longrightarrow}\limits^{{X}_{3}}_{\begin{array}{c}tertiary\\ degrader\end{array}}\ldots$$

Again, all the kinetic, stoichiometric and transport parameters were assumed identical for all three substrates and three biomass types, taking the values from *Case A1*.

#### B. Aerobic biofilms (organic electron donor, oxygen as electron acceptor)

##### Case B1: aerobic heterotrophic cross-feeding

In case of aerobic biofilms, where a complex organic substrate is degraded, it is common that conventional heterotrophs fully oxidize the substrate instead of dividing labor with different microbial groups organized in strata. Then the question is why in aerobic environments division of labor is not favored. Several hypotheses have been presented^34^, but none based on the role of diffusion of intermediates. To evaluate the role of substrate and oxygen diffusion in aerobic biofilms, we used the same cross-feeding reaction scheme, but with growth rate of both microbial species function of the dissolved oxygen concentration too (double Monod limitation) (Eq. (4), Table 2). Notably, the stoichiometry of growth processes is also changed to respect the COD balance including now O_2_ as electron acceptor, i.e., leading to less production of secondary substrate *S*_2_.

4 $$S_{1}\mathop{\longrightarrow}\limits^{X_{1}}_{O_{2}^{\nearrow}\begin{array}{c}primary\\ degrader\end{array}}\, S_{2}\mathop{\longrightarrow}\limits^{X_{2}}_{O_{2}^{\nearrow}\begin{array}{c}secondary\\ degrader\end{array}} {\ldots}$$

For the aerobic case, the so-called oxygen utilization yield on substrate (*a*, with units gO2/gCOD-S) that quantifies the oxygen dependency of COD conversion by aerobic heterotrophic bacteria (Table 2) has relevance. A higher *a* value means more oxygen is required to degrade a given amount of organic matter (COD). This suggests a more oxygen-intensive metabolism. A lower *a* value indicates that the process may involve more efficient oxygen utilization or alternative pathways (e.g., partial anoxic degradation).

##### Case B2: aerobic autotrophic cross-feeding

In the case of cross-feeding autotrophs growing on aerobic biofilms (e.g., canonical nitrification) the primary substrate used for energy is not the main carbon source for growth, which creates a different scenario, because the use of the primary substrate do not reduce the availability of the secondary substrate, as it occurs in the aerobic cross-feeding biofilms (see Table 2). Nitrifying biofilms were reported to present stratification, with external layers occupied by ammonia oxidizing bacteria and nitrite oxidizing bacteria relegated to inner layers, which easy the production of nitrite in biofilm reactors for the treatment of wastewater^35^. Consequently, the case of aerobic autotrophic cross-feeding biofilms was also explored (see detailed description in Table 2).

### Parameter values and simulation cases

#### Base case

For a schematic representation of the model and base case parameters, see Fig. 8. To keep the model evaluation simple, the diffusion coefficients of primary substrate and intermediate in the biofilm were set equal (${D}_{S1}={D}_{S2}=$10^−4^ m^2^/d). In such way, the resultant spatial organization would be uniquely ruled by the distinct access to substrate by each of the microbial species. The concentrations in the inflow were set to *S*_*in,*1_ = 100 and *S*_*in,*2_ = 0 gCOD/m^3^, with a reactor volume *V*_*b*_ = 1 m^3^ and biofilm area *A*_*f*_ = 100 m^2^, all kept constant. However, the influent flowrate *Q*_*b*_ was varied to regulate the bulk liquid concentration for *S*_1_ to a value of *S*_*b,*1_ = 10 gCOD/m^3^ (as done in ref. ^45^). In this way, the conversion of *S*_1_ was fixed to 90%, while the bulk water concentration of *S*_2_ is actually a model output. The biofilm model needs a total biomass concentration, *X*_*f,tot*_, taken as 100 kgCOD/m^3^, and a biofilm thickness assumed here *L*_*f*_ = 300 μm. The choice of initial values (concentrations of substrates in biofilm and liquid, biomass in biofilm) do not alter the obtained steady states, i.e., no multiplicity of solutions was detected. When exploring the effects of changing the bulk substrate concentration *S*_b,1,_ the inflow concentration of *S*_1_ (*S*_in,1_) was always kept constant at 100 gCOD/m^3^ and the flow rate *Q*_*b*_ was changed. All model parameters are listed in Supplementary Table 1.

#### Quantification of the apparent growth yield disproportion

We have defined the ratio of secondary to primary degrader amounts retained in the biofilm (i.e., the dimensionless *m*_2_/*m*_1_, with amounts *m*_*i*_ defined in the Supplementary Information) as a quantification of the apparent growth yield disproportion. Since the growth yields ratio *Y*_2_/*Y*_1_ equals 1 (same stoichiometry, see Table 2), any value of the ratio *m*_2_/*m*_1_ larger than 0.9 is considered here as an apparent growth yield disproportion.

## Supplementary information

Supplementary Information

## Data Availability

The COMSOL model can be made available upon request.
