# Supplementary material for: Diffusion-based mechanism explains spatial organization in cross-feeding biofilms
Source: NPJ Biofilms Microbiomes. 2025 Jun 11;11:102. doi: 10.1038/s41522-025-00719-5 (PMC12159163; doi:10.1038/s41522-025-00719-5)
Supplement: Supplementary file 1 — Supplementary Information [file 41522_2025_719_MOESM1_ESM.pdf]

SUPPLEMENTARY INFORMATION

Diffusion-based mechanism explains spatial organization in cross-feeding biofilms

Julio Pérez[1,*], Cristian Picioreanu[2]

[1] Department of Chemical, Biological and Environmental Engineering, Universitat Autònoma de Barcelona; Campus UAB, 08193 Bellaterra (Barcelona), Spain

[2] Biological and Environmental Science and Engineering Division (BESE), King Abdullah University of Science and Technology (KAUST), Thuwal 23955-6900, Saudi Arabia

*Corresponding author: Julio.Perez@uab.es

Content:

Supplementary Note 1

Supplementary Table 1

Supplementary Table 2

Supplementary Figure 1

Supplementary Figure 2

Supplementary Figure 3

Supplementary Figure 4

Supplementary Figure 5

Supplementary Note 1: Model Description

The one-dimensional biofilm model is based on the traditional approach formulated by Wanner and Gujer (1986), which also formed the base for the *Aquasim* software implementation Wanner and Reichert (1996). However, we implemented the mathematical model in a more modern environment, *COMSOL Multiphysics 6.2* (www.comsol.com). The biofilm phase is in contact with an aqueous phase, representing a continuous ideally mixed biofilm reactor. Following the well-accepted IWA methodology in modelling environmental engineering applications (Hermann et al., 2006; Henze et al., 2000), the system contains two types of components with different physics: soluble components, S, and particulate components, X. Balances are set up for these two types of components in the biofilm phase and in an aqueous phase (called also "bulk liquid").

Mass balances in the biofilm phase

It is assumed that concentration gradients for both solutes and particulates develop only in one direction in the biofilm, direction *z* normal to the support material (thus, from the biofilm base to the biofilm interface with water). The time-dependent balance for soluble components in the biofilm can be expressed function of concentration of any solute *i*, S_i, as state variable. The balance includes traditionally solute transport by diffusion only and source terms representing the reaction rates:

$$\frac{\partial S_i}{\partial t} = D_i \frac{\partial^2 S_i}{\partial z^2} + R_i \qquad \text{(S1)}$$

with initial condition $S_i\big|_{t=0,z} = S_i^0$ and boundary conditions $\left.\frac{\partial S_i}{\partial z}\right|_{t,z=0} = 0$ and $S_i\big|_{t,z=L_f} = S_{b,i}\big|_t$. D_i is the diffusion coefficient of solute *i* (S_1, S_2, O_2, etc.) in the biofilm, normally a fraction of the value in water because of hindered diffusion in the biofilm matrix, and considered here independent of position in the biofilm. R_i is the net volumetric reaction rate at any position *z* in the biofilm ($z \in \left[0, L_f\right]$) in (kg/m^{3}d). At initial time, the concentration of solute *i* is S_i^0 at any position in the biofilm. The most typical boundary conditions imply no flux of solutes at the biofilm base (at *z*=0)

and known solute concentration at the biofilm surface set as the value in the bulk liquid (at $z=L_f$), at any moment in time. The dissolved oxygen balances, in the model cases with aerobic biofilms, are similar with those for the substrates. Again, to simplify the model analysis, the external mass transfer resistance for solutes was neglected (no diffusion boundary layer).

The balance for particulates considers transport by convection and source terms due to biomass growth or decay, with state variable the concentration X_i:

$$\frac{\partial X_i}{\partial t} = -\frac{\partial\left(u_f X_i\right)}{\partial z} + R_i \qquad \text{(S2)}$$

with initial condition $X_i\big|_{t=0,z} = X_i^0$ and boundary condition $\left.\frac{\partial X_i}{\partial z}\right|_{t,z=0} = 0$. R_i is the net volumetric reaction rate of biomass generation or loss at any position z in the biofilm in (kg/m^{3}d). It is essential that some biomass exists in the biofilm at initial time, meaning that the concentration of particulate i is X_i^0 typically chosen as uniform biomass distribution throughout the biofilm. $u_f(z)$ is the advective velocity of particulates at any position in the biofilm, which is a result of the fact that produced particulate biomass at a certain position z in the biofilm pushes all the biomass between that position and the biofilm surface L_f. Thus, u_f is a cumulative velocity, typically increasing from z=0 to L_f. In order to compute u_f, several simplifying assumptions can be made. Here we assume that the total biomass concentration in the biofilm remains constant, thus implying that a growing particulate concentration has the effect of decreasing another particulate concentration at that position (the total biomass concentration in the biofilm is conserved both in space and time). When summing biomass balances for all components i and apply the chain differentiation for the convective rate of biomass transport one obtains $\frac{\partial \sum X_i}{\partial t} = -u_f \frac{\partial \sum X_i}{\partial z} - \sum X_i \frac{\partial u_f}{\partial z} + \sum R_i$. With the assumption that $\sum X_i = X_{tot}$ is constant in space and time, one obtains:

$$\frac{du_f}{dz} = \frac{\sum R_i}{X_{tot}} \qquad \text{(S3)}$$

with boundary condition $u_f\big|_{t,z=0} = 0$ (i.e., fixed biomass at the base).

Mass balances in the aqueous phase (bulk liquid)

It is assumed that the bulk liquid in which the biofilm develops is ideally mixed, and there is continuous operation (feed/discharge) at constant volume. The time-dependent balance for soluble components in the bulk liquid can be expressed function of concentration of any solute *i*, $S_{b,i}$, as state variable:

$$\frac{dS_{b,i}}{dt} = \frac{Q_b}{V_b}\left(S_{in,i} - S_{b,i}\right) + J_{f,i}\frac{A_f}{V_b} \tag{S4}$$

with initial condition $S_{b,i}\big|_{t=0} = S_{b,i}^0$. Q_b is the volumetric inflow/outflow rate (m^3/d), V_b is the bulk liquid volume (m^3), $S_{in,i}$ is the concentration of a solute in the inflow (kg/m^3), A_f is biofilm surface area in contact with water (m^2), $J_{f,i}$ is flux of solute exchanged between water and biofilm (kg/m^2d), here only diffusive $J_{f,i} = -D_i \frac{dS_i}{dz}\bigg|_{t,z=Lf}$. To avoid interference from microbial activity in the bulk liquid when identifying mechanisms and principles, we assumed that the suspended biomass does not contribute significantly to the overall substrate conversion in the reactor. Thus, no substrate reaction rate in the bulk liquid and no suspended biomass concentrations.

Coupled model

The overall model involves solving simultaneously equations (S1) to (S4) with their associated initial and boundary conditions. One obtains: solute $S_{b,i}(t)$ concentrations in the bulk liquid in time; solute $S_i(t,z)$ and particulate $X_i(t,z)$ concentrations along the biofilm thickness and in time; and the additional variable $u_f(t,z)$.

The reaction system has to be defined according to the microbial interaction case. See Table 1 in the main text.

Model parameters

Since there was no intention to mimic any specific process, the parameter values were rounded to simple numbers. Surely, the parameter values would change model outputs, but conclusions and mechanisms - which are here the main goal of the simulations - remain unaltered. A continuous biofilm reactor with a capacity of V_b=1 m^3 and a flat area for biofilm development of A_f=100 m^2 were arbitrarily used. The substrate concentration in the bulk liquid was set by regulation (proportional control) of the flow rate Q_b and setting $S_{in,1}$=100 g/m^3. This simulation strategy allows to explicitly investigating the effect of substrate concentration in the bulk liquid. All material units here are expressed as mass of COD (chemical oxygen demand) or O_2, thus consistent with bioenergetic balances. A biofilm thickness of L_f=300 µm was set, so that there is almost complete depletion of substrates in the biofilm depth, but not "too much" inert zone. Equal concentrations of *active* biomass types were set initially, with a total concentration X_{tot}=100 kgCOD·m^{-3}. Diffusion coefficients of substrate, intermediates and oxygen in the biofilm were all equal to 10^{-4} m/d, unless otherwise stated. For the aerobic case, an oxygen concentration in the liquid of 6 mgO$_2$/L was set, except otherwise stated.

The stoichiometry and kinetics used are in **Table** 1 (main text). The model parameters are summarized in **Supplementary Table** 1. The simulations were run for at least 1000 days, with biomass profiles inspected to assure that the results reached steady state. Initial values for the simulations did not alter the results at steady state (i.e., there was no known multiplicity of steady states).

Performance indicators

Biomass retention m_i was defined as the biomass amount (kg) existing in the reactor at stationary state. For the chemostat: $m_i = X_{b,i} V_b$ with V_b the bulk liquid volume (m^3) and $X_{b,i}$ the concentration (kg/m^3) of suspended biomass type *i*, computed by solving the coupled mass balances for substrates (S_1, S_2) and biomasses (X_1, X_2) in the chemostat. For the biofilm reactor: $m_i = A_f \int_0^{L_f} X_{f,i} dx$ with A_f the biofilm area and $X_{f,i}$ the concentration of biomass type *i* in the biofilm.

The degree of conversion of the primary substrate is $x_1 = \frac{S_{in,1} - S_{b,1}}{S_{in,1}}$, while for the secondary substrate it is $x_2 = \frac{(1-Y_1)(S_{in,1} - S_{b,1}) - S_{b,2}}{(1-Y_1)(S_{in,1} - S_{b,1})}$ because (1-Y_1) g of S_2 is formed from 1 g of S_1. These definitions hold both for chemostat and for biofilm reactor.

Substrate-consumption specific biomass retention was calculated as $\frac{1}{q_{S,i}} = \frac{m_i}{A_f \int_0^{L_f} R_{Si} dz}$ for biofilm reactors, with biomass retention m_i (kg), biofilm surface area in contact with water A_f (m^2), and the rate of substrate i consumed by biomass i which is $\frac{1}{Y_i} \mu_{m,i} \frac{S_i}{K_{S,i} + S_i} X_i$ (kg/m^3s). For chemostats, this is simply $\frac{1}{q_{S,i}} = \frac{1}{\frac{1}{Y_i} \mu_{m,i} \frac{S_i}{K_{S,i} + S_i}}$.

References

Batstone D.J. et al. The IWA Anaerobic Digestion Model No. 1 (ADM1) (IWA Publishing, 2002).

Henze M., Gujer W., Mino T. & van Loosdrecht M. Activated sludge models ASM1, ASM2, ASM2D and ASM3 (IWA Publishing, 2000).

Hermann E. et al. Mathematical Modeling of Biofilms (IWA Publishing, 2006).

Wanner O. & Gujer W. A multispecies biofilm model. *Biotechnol Bioeng.* **28**, 314-328 (1986).

Wanner O. & Reichert P. Mathematical modeling of mixed-culture biofilms. *Biotechnol Bioeng.* **49**, 172-184 (1996).

Supplementary Table 1. Model parameters. Where a parameter contains the subscript *i*, this means equal values for either primary/secondary degrader microorganisms or for their respective substrates.

Parameter description	Symbol	Value	Comments [Source]
Biofilm thickness	L_f	300 μm	A reasonable thickness allowing full substrate utilization
Biomass concentration in the biofilm	X_{tot}	100 gCOD-X L^{-1}	In the range for anaerobic biofilms [Hermann et al., 2006]
Diffusion coefficient substrate in the biofilm	D_i	$1 \cdot 10^{-4}$ m^2 d^{-1}	Typical for small molecules in water at 25-35 °C
Biomass yield from substrate	Y_i	0.1 gCOD-X gCOD-S^{-1}	Assumed as in anaerobic digestion [Batstone et al., 2002]
		0.2 gCOD-X gN^{-1}	For aerobic nitrification case [Henze et al, 2000]
Oxygen utilization yield on substrate	a_i	0.5 gO_2 gCOD-S^{-1}	Reasonable for heterotrophs [Henze et al, 2000]
Oxygen demand for ammonium and nitrate	α_1<br>α_2	3.45 gO_2 gN^{-1}<br>1.15 gO_2 gN^{-1}	Nitrification: ammonium to nitrite (1) and nitrite to nitrate (2) [Henze et al, 2000]
Maximum specific biomass growth rate	$\mu_{m,i}$	1 d^{-1}	Within the acceptable range for anaerobic digestion [Batstone et al., 2002]
Decay rate coefficient	b_i	0.01 d^{-1}	Slow microbial decay rate [Batstone et al., 2002]
Half-saturation (Monod) coefficient for substrate	$K_{S,i}$	1 gCOD-S m^{-3}	Typical value [Batstone et al., 2002]
Half-saturation (Monod) coefficient for oxygen	$K_{O2,i}$	0.1 gO_2 m^{-3}	Typical value [Henze et al, 2000]

Reactor bulk liquid volume	V_b	1 m^3	Arbitrary
Reactor biofilm area	A_f	0.1 m^2	Arbitrary, typical for bioreactors with reduced biofilm content
Substrate concentration in inlet	$S_{in,1}$	100 gCOD-S m^{-3}	Typical value for primary substrate (i=1), and no secondary/tertiary substrates (i=2,3) in influent
Substrate concentration in bulk liquid	$S_{b,1}$	10 gCOD-S m^{-3}	Set value for the primary substrate, controlled by changing the influent flow rate Q_b
Oxygen concentration in bulk liquid	$S_{b,O2}$	6 gO_2 m^{-3}	Fixed, as for a well-aerated biofilm reactor

Supplementary Table 2. Stoichiometry matrix and rates used in the anaerobic degradation of butyrate (S_1) to acetate (S_2') and hydrogen (S_2") by organism X_1, followed by methanogenesis by aceticlastic (X_2') and and hydrogenotrophic (X_2") organisms. Most of the stoichiometric and rate parameters were taken from the anaerobic digestion model (ADM1, Batstone et al., 2002): Y_1=0.06 gCOD-X/gCOD-butyrate, Y_2'=0.05 gCOD-X/gCOD-acetate, and Y_2''=0.06 gCOD-X/gCOD-H_2; maximum specific rates $\mu_{m,1}$=1.2 1/d, $\mu_{m,2}'$=0.4 1/d, and $\mu_{m,2}''$=2.1 1/d; all decay rate coefficients equal, b = 0.02 1/d. However, the half-saturation coefficients were taken equal K = 1 gCOD/m^3, the value from the general DEMO model and smaller than in ADM1 (Batstone et al., 2002) to counteract the diffusion effects. The diffusion coefficients were $0.87 \cdot 10^{-9}$ m^2/s (butyrate), $1.1 \cdot 10^{-9}$ m^2/s (acetate), $5 \cdot 10^{-9}$ m^2/s (H_2) and $1.5 \cdot 10^{-9}$ m^2/s (CH_4).

Component →	S_1 (But)	S_2' (Ac)	S_2'' (H2)	P (CH_4)	X_1	X_2'	X_2'	X_I	Rate
Process ↓	primary substrate (butyrate)	secondary substrate 1 (acetate)	secondary substrate 2 (hydrogen)	end product (methane)	primary degrader (acetogen)	secondary degrader 1 (aceticlastic)	secondary degrader 2 (H-troph)	inert biomass	
1. Growth X_1	$-\frac{1}{Y_1}$	$0.8\frac{1-Y_1}{Y_1}$	$0.2\frac{1-Y_1}{Y_1}$		1				$\mu_{m,1}\frac{S_1}{K_S+S_1}X_1$
2. Decay X_1					-1			1	$b\,X_1$
3. Growth X_2'		$-\frac{1}{Y_2'}$		$\frac{1-Y_2'}{Y_2'}$		1			$\mu_{m,2}'\frac{S_2'}{K_S+S_2'}X_2'$
4. Decay X_2'						-1		1	$b\,X_2'$
3. Growth X_2''			$-\frac{1}{Y_2''}$	$\frac{1-Y_2''}{Y_2''}$			1		$\mu_{m,2}''\frac{S_2''}{K_S+S_2''}X_2''$
4. Decay X_2''							-1	1	$b\,X_2''$
Units	$\frac{g\,COD}{m^3}$	$\frac{g\,COD}{m^3}$	$\frac{g\,COD}{m^3}$	$\frac{g\,COD}{m^3}$	$\frac{g\,COD}{m^3}$	$\frac{g\,COD}{m^3}$	$\frac{g\,COD}{m^3}$	$\frac{g\,COD}{m^3}$	$\frac{g\,COD}{m^3 d}$

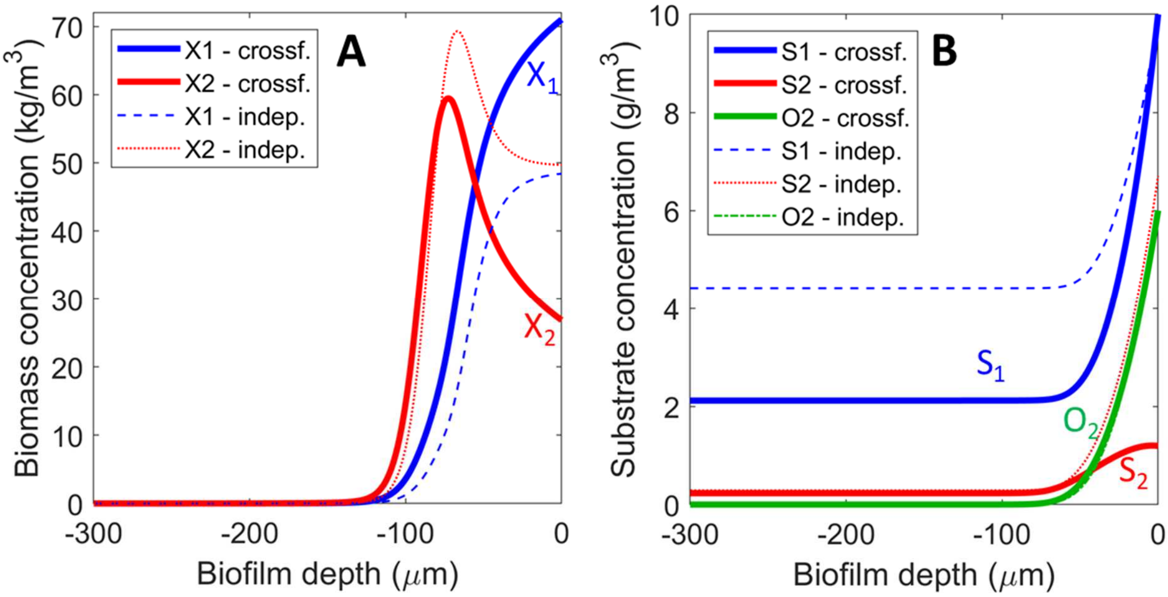

Supplementary Figure 1. Aerobic heterotrophic case (*Case B1*) with better oxygen affinity of X_2 (i.e., $K_{O2,2}$= 0.25·$K_{O2,1}$) comparing cross-feeding and independent cases. Emerging (i.e., steady state) active biomass (**A**) and substrate (**B**) concentration profiles over the biofilm depth (water interface is at 0 μm).

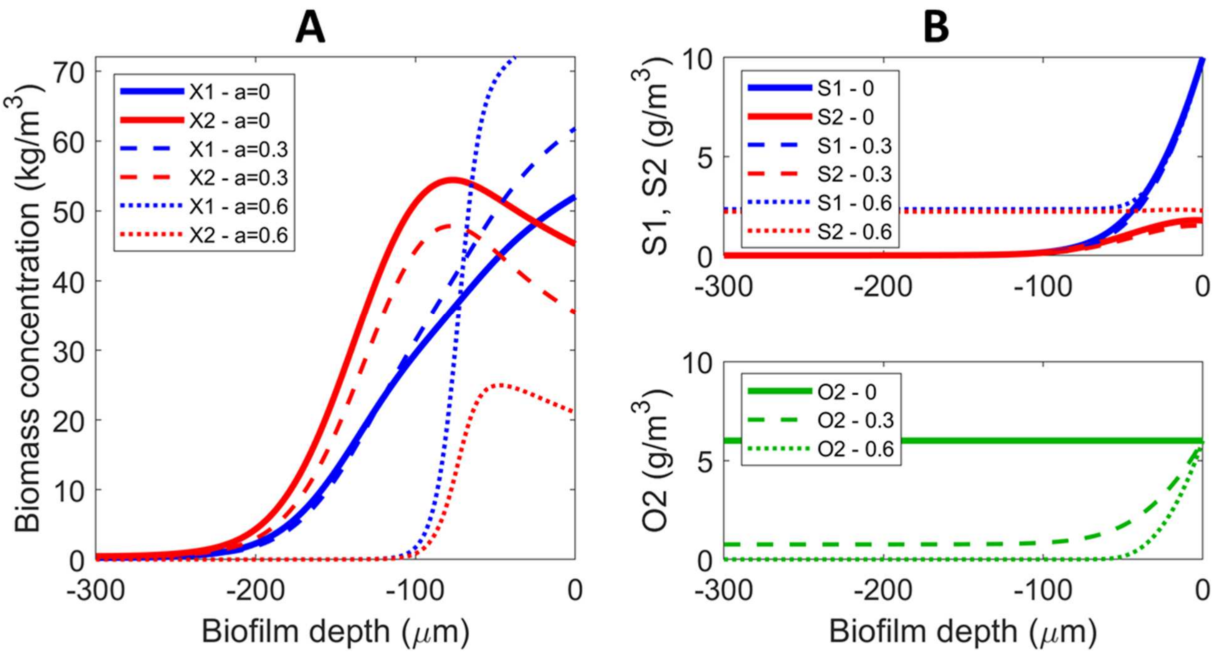

Supplementary Figure 2. Effect of the yield on oxygen, *a*, on the distribution in the biofilm of (**A**) biomass (**B**) solutes, for the aerobic heterotrophic cross-feeding (*Case B1*).

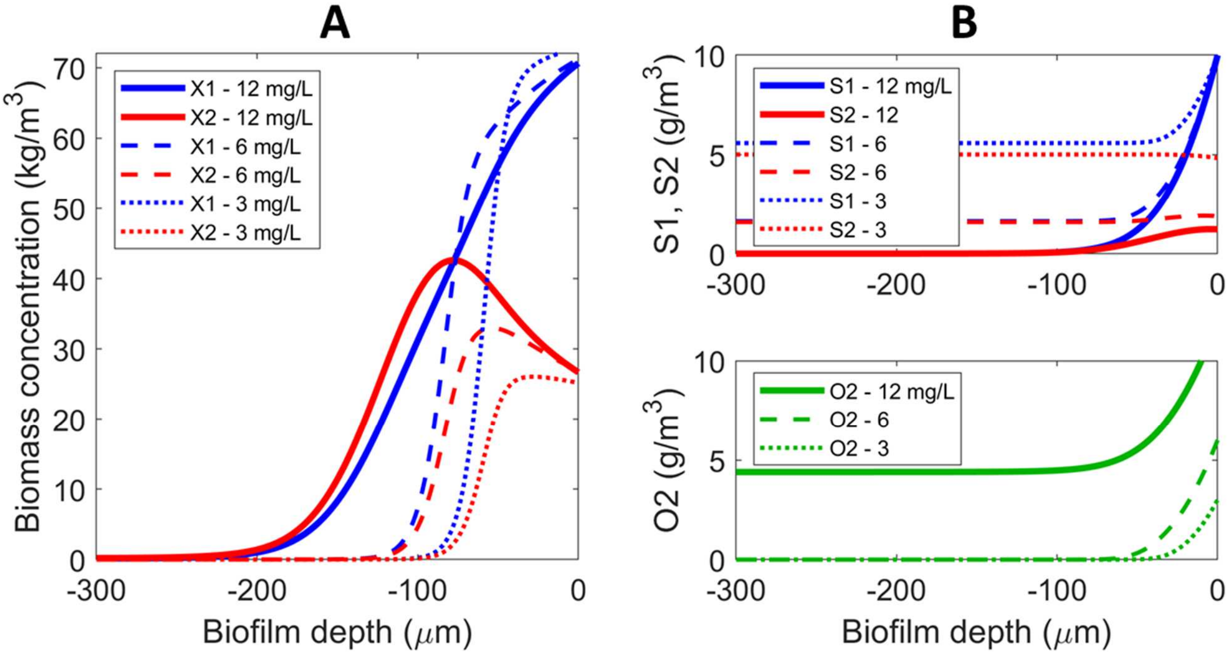

Supplementary Figure 3. Effect of the dissolved oxygen concentration in the water on the distribution in the biofilm of (**A**) biomass (**B**) solutes, for the aerobic heterotrophic cross-feeding (*Case B1*).

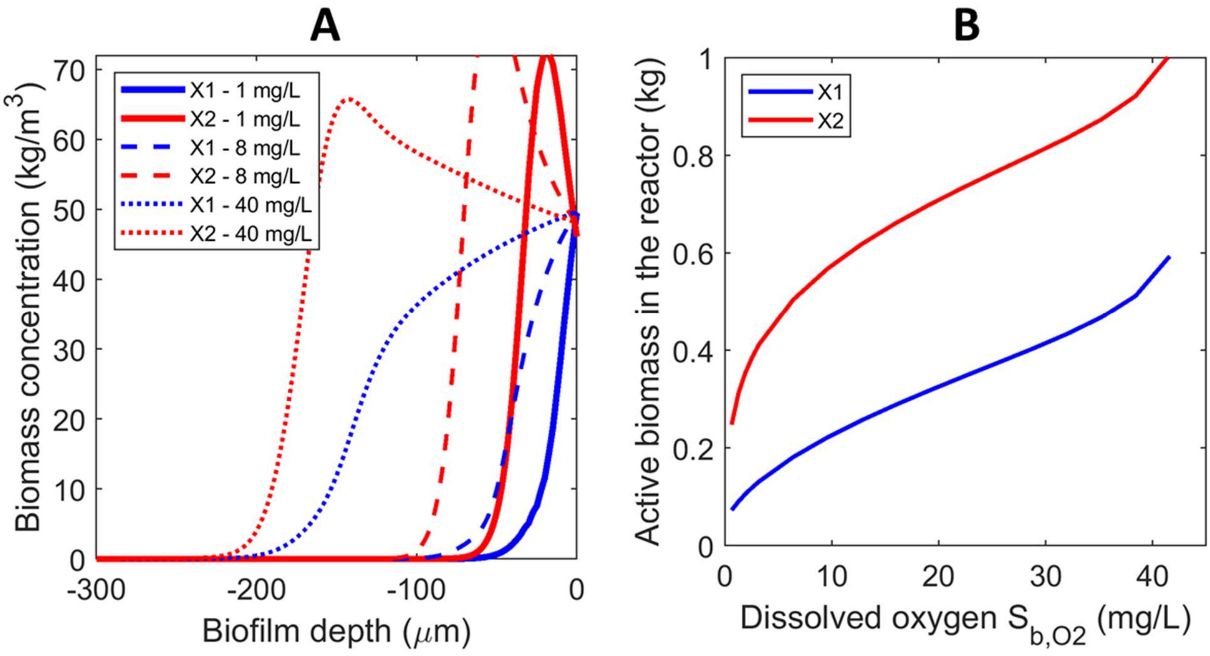

Supplementary Figure 4. Aerobic autotrophic cross-feeding biofilm (*Case B2*) with better oxygen affinity of X_2 (i.e., $K_{O2,2}$= 0.25·$K_{O2,1}$). (**A**) distribution of biomass in the biofilm; (**B**) active biomass in the reactor function of dissolved oxygen concentration in the bulk liquid.

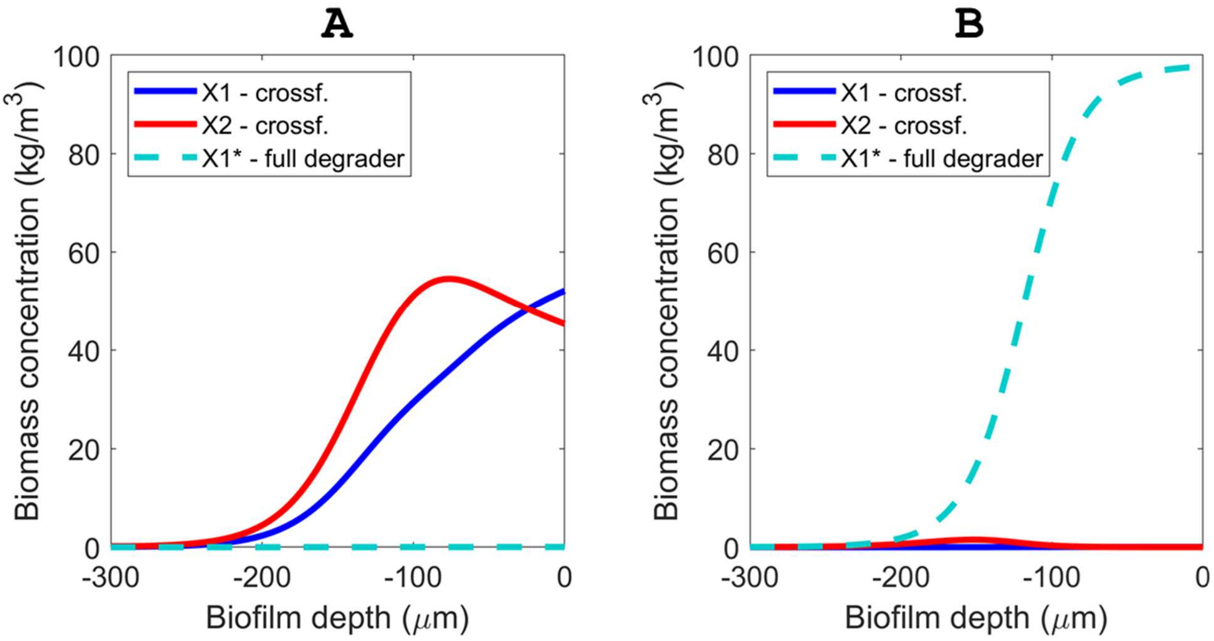

Supplementary Figure 5. Competition between the cross-feeding consortium $X_{1/}$ X_2 and a full degrader (X_1^*). Steady state microbial distribution for a biofilm for a full degrader (X_1^*) exhibiting marginally smaller maximum specific growth rate than that of the primary degrader (X_1), $\mu_{m,1}^* = 0.95\mu_{m,1}$ (**A**) or a marginally larger maximum specific growth rate $\mu_{m,1} = 0.95\mu_{m,1}^*$ (**B**).
